# Cavitation Enhancing Nanodroplets Mediate Efficient DNA Fragmentation in a Bench Top Ultrasonic Water Bath

**DOI:** 10.1371/journal.pone.0133014

**Published:** 2015-07-17

**Authors:** Sandeep K. Kasoji, Samantha G. Pattenden, Ewa P. Malc, Chatura N. Jayakody, James K. Tsuruta, Piotr A. Mieczkowski, William P. Janzen, Paul A. Dayton

**Affiliations:** 1 Joint Department of Biomedical Engineering, University of North Carolina, Chapel Hill, North Carolina, and North Carolina State University, Raleigh, North Carolina, United States of America; 2 Center for Integrative Chemical Biology and Drug Discovery, Eshelman School of Pharmacy, University of North Carolina, Chapel Hill, North Carolina, United States of America; 3 Department of Genetics, High Throughput Sequencing Facility, University of North Carolina, Chapel Hill, North Carolina, United States of America; 4 Department of Pediatrics, University of North Carolina, Chapel Hill, North Carolina, United States of America; University of Maryland School of Medicine, UNITED STATES

## Abstract

A perfluorocarbon nanodroplet formulation is shown to be an effective cavitation enhancement agent, enabling rapid and consistent fragmentation of genomic DNA in a standard ultrasonic water bath. This nanodroplet-enhanced method produces genomic DNA libraries and next-generation sequencing results indistinguishable from DNA samples fragmented in dedicated commercial acoustic sonication equipment, and with higher throughput. This technique thus enables widespread access to fast bench-top genomic DNA fragmentation.

## Introduction

Next-generation sequencing is an attractive technology for detecting genetic disorders and characterizing the underlying molecular signature of disease to determine therapeutic options. It is not yet, however, a routine diagnostic tool due to a lack of standardization in DNA sample preparation, cost, and difficulties in data interpretation [1, 2]. Specifically, random, unbiased fragmentation of DNA is a bottleneck in next-generation sequencing sample preparation pipelines due to serial processing of samples, cost limitations, poor DNA sample quality, and lack of reproducibility of fragment size between sample types [3, 4].

Methods for fragmentation of DNA include enzymatic digestion, nebulization, hydrodynamic shearing, and sonication. Enzymatic digestion using DNase I, micrococcal nuclease, or restriction enzymes is very efficient, but introduces an enzyme bias. Regions of transcriptionally silent, tightly packed (heterochromatic) DNA and DNA with high G-C content can be refractive to enzymatic digestion and many enzymes only create nicks in the DNA instead of cutting completely though both strands [5–11]. The nebulization process shears solubilized DNA by forcing it through a pressurized nozzle (atomization). This method is fast, but requires large quantities of DNA and often results in a large distribution in the DNA fragment size and cross-contamination between samples [12–14]. Hydrodynamic shearing involves forcing solubilized DNA through a mesh. It has the advantage of rapidly producing small DNA fragments of nearly uniform length. This method, however, is costly and the screen used for shearing is prone to clogging and cross-contamination between samples [15, 16]. Finally, acoustic sonication uses ultrasound to mechanically shear DNA by cavitation. This method typically produces inconsistent results and is time consuming since DNA extracted from cells or tissue must be optimized each time to ensure that fragmentation occurs to the desired size range [3, 17]. Also, similar to enzymatic digestion, heterochromatic DNA or DNA with high G-C content are very difficult to shear, which creates a bias toward better shearing efficiency in euchromatic and A-T rich regions [3, 18–20]. Dedicated high-intensity focused sonicators such as the Covaris Adaptive Focused Acoustics instrument, or the lower-intensity Diagenode Bioruptor instrument, produce more consistent DNA fragmentation results than a single probe sonication device, however they can be financially inaccessible for many laboratories.

We investigated whether a biologically inert agent could be added to DNA samples to amplify cavitation in a sonication device and improve the consistency and speed of genomic DNA fragmentation. We initially tested lipid-encapsulated microbubbles, which are bubbles that are typically in the 1–10 micron (μm) diameter size range, and have been used in medical diagnostics as contrast agents for ultrasound imaging for approximately two decades [21]. The highly compressible core of a gas filled microbubble enables it to compress and expand in a pressure field in a process called cavitation [22, 23]. High-speed photography has shown that the microbubble shell can expand and contract on the order of 700 meters per second even at only moderate acoustic pressures (1.2 MPa, 2.4 MHz) [24]. At even higher acoustic pressures, which are typical of a commercial sonicator, microbubble cavitation is very intense and results in a violent collapse, locally releasing a great amount of mechanical energy.

As an alternative to microbubbles, we designed a novel formulation of phase-change nanodroplets [25, 26]. These phase-change nanodroplets are composed of a volatile, liquid perfluorocarbon core stabilized with a phospholipid monolayer shell with a diameter on the order of 200–400 nm [27]. These excipients are biologically inactive when combined with genomic DNA samples. When subject to sufficient acoustic energy and temperature, the nanodroplets vaporize into microbubbles that have approximate diameters of 1–5 μm. The phase-change nanodroplets are more stable than microbubbles and require energy to vaporize and then require additional energy to cavitate, therefore prolonging the cavitation enhancing effect compared to bubbles alone. The increased stability of nanodroplets compared to microbubbles has been demonstrated by Sheeran *et*. *al*. in an *in vivo* animal model circulation time study [28]. We show that nanodroplets perform as an effective cavitation enhancement agent for the fragmentation of genomic DNA (gDNA), decreasing sonication time while preserving DNA fragment size distribution and yield.

## Methods

### Genomic DNA preparation

gDNA was prepared from a 10 mL saturated culture of the *Saccharomyces cerevisiae* wild type haploid strain, BY4741 (MAT**a** *his3Δ1 leu2Δ0 met15Δ0 ura3Δ0*). gDNA was purified using the MasterPure Yeast DNA Purification Kit (Epicenter, Madison, WI, USA #MPY80200), according to manufacturer instructions. Quantification of purified DNA was performed on a Qubit 2.0 Fluorometer.

### Nanodroplet Preparation

Microbubbles consisting of a gas core and an encapsulating lipid monolayer were generated by mechanical agitation of a lipid solution in the presence of decafluorobutane gas (DFB). The lipid solution was composed of 1,2-distearoyl-sn-glycero-3-phosphocholine (DSPC) (Avanti Polar Lipids, Alabaster, AL, USA) stabilized by 1,2-distearoyl-sn-glycero-3-phosphoethanolamine-N-methoxy (polyethylene-glycol)-2000 (DSPE-PEG2000) (Avanti Polar Lipids, Alabaster, AL, USA) in a 9 to 1 molar ratio. The lipid solution was formulated with phosphate-buffered saline solution containing propylene glycol (15% v/v) and glycerol (5% v/v) at a final lipid concentration of 1.0 mg/mL. 1.5 mL aliquots of the lipid emulsion were dispensed into 3 mL vials and sealed with a butyl rubber septum. The air-filled headspace in the vial was exchanged with decafluorobutane (C_4_F_10_, boiling point: -2°C) gas (Fluoromed, Round Rock, TX, USA). The vial was vigorously shaken for 45 seconds using a high-speed mixer (Vialmix, Bristol-Myers Squibb Medical Imaging, North Billerica, MA, USA) to form the microbubble solution. The solution contained approximately 10^10^ bubbles/mL with a diameter of 1.07 ± 0.9 μm, measured with an Accusizer 780 (Particle Sizing Systems, Santa Barbara, CA, USA).

Nanodroplets were formed by cooling and compressing the microbubble solution to induce a gas-to-liquid phase transition of the decafluorobutane gas. Dry ice was used to cool approximately 100 mL of 2-propanol to -9°C in a beaker. A 20-gauge needle connected to a fully-drawn 60 mL syringe was inserted into the DFB-headspace through the butyl rubber septum of the vial. The vial was then submerged in the chilled 2-propanol, and swirled while simultaneously increasing the pressure within the vial by depressing the syringe plunger, eventually condensing the bubbles into droplets. The nanodroplet solution was stored at -20°C when not in use. 20 μL of nanodroplets were dispensed in each DNA sample for sonication.

### DNA Fragmentation: Covaris E110 Sonicator

Five μg of BY4741 gDNA was analyzed in duplicate in either Covaris microTUBEs (Covaris, Woburn, MA, USA #520045), or borosilicate glass vials in a final volume of 100 μL in TE (10 mM Tris-HCl, pH 8.0, 1 mM EDTA). 10 μL of nanodroplets were added to the borosilicate glass vials. Sample tubes were submerged up to the cap in a water bath and sonicated for 2 minutes each at 20% duty cycle, Intensity 8, and 200 cycles per burst. After fragmentation, 20 μL of DNA was combined with 10X loading buffer (50% glycerol with Orange G and SYBR green) and loaded onto a 1.5% agarose gel and subjected to gel electrophoresis. For high throughput sequencing preparation, each sample was concentrated in a Zymo Research ChIP DNA Clean & Concentrator column (Zymo Research, Irvine, CA, USA #D5201), followed by quantification on a Qubit 2.0 Fluorometer (Life Technologies, Grand Island, NY, USA). DNA fragment quality and size was assessed using an Agilent D1000 ScreenTape system (Agilent Technologies, Santa Clara, CA, USA).

### DNA Fragmentation: Branson Sonifier Bath

Five μg BY4741 gDNA samples were analyzed in duplicate using 0.2 mL PCR strip tubes (Genesee Scientific, Morrisville, NC, USA #27–104) in 150 μL of TE (10 mM Tris-HCl, pH 8.0, 1 mM EDTA). Samples received either 10 μL of nanodroplets or 10 μL of TE. Tubes were placed in a linear acrylic holder (0.5” spacing) and positioned in the center of the ultrasonic bath so that the sample tubes were submerged up to the cap in water. The water bath was either cooled to 3–4°C using a circulating refrigerated bath chiller followed by sonication for 5 minutes, or chilled ice water was added just prior to sonication. Following fragmentation, 20 μL of DNA was combined with 10X loading buffer (50% glycerol with Orange G and SYBR green) and loaded onto a 1.5% agarose gel and subjected to gel electrophoresis. Densitometry was performed using Image J software. For high throughput sequencing preparation, each sample was concentrated in a Zymo Research ChIP DNA Clean & Concentrator column (Zymo Research, Irvine, CA, USA #D5201), followed by quantitation on a Qubit 2.0 Fluorometer. (Life Technologies, Grand Island, NY, USA) DNA fragment quality and size was assessed using an Agilent D1000 ScreenTape system (Agilent Technologies, Santa Clara, CA, USA).

The presence of cavitation and the enhancement of cavitation with nanodroplets in both the Covaris and Branson ultrasonic bath were confirmed using a starch-potassium iodide test (S1 Protocol).

### Next-generation Sequencing

Yeast gDNA samples sonicated in the Covaris E110 were prepared using Kapa DNA Library Prep Kit for Illumina (Illumina, San Diego, CA, USA #8234). Libraries were prepared using both agarose gel size selection (Sage Science Pippin Prep Targeted DNA Size Selection System, Sage Science, Inc., Beverly, MA, USA) and magnetic bead selection (Magbio HighPrep PCR beads #AC-60500), with 0.45 and 0.2 ratios for the first and second step of size selection, respectively, Magbio Genomics, Gaithersburg, MD, USA) to ensure that the size selection method did not influence sequencing result comparison.

Yeast gDNA samples fragmented in the ultrasonic bath were prepared using the Illumina TruSeq Nano DNA LT Sample Preparation Kit (Illumina, San Diego, CA, USA #FC-121-4001). Each sample was prepared with either 200 ng or 100 ng of DNA and size-selected using magnetic beads as described.

Following preparation, all libraries were quantified using a Qubit 2.0 Fluorometer (Life Technologies, Grand Island, NY, USA). DNA fragment quality and size was assessed using Bio-Rad Experion Automated Electrophoresis System (Bio-Rad Laboratories, Inc., Hercules, CA, USA).

Sequencing data have been deposited to the BioProject database (National Center for Biotechnology Information), BioProject accession PRJNA284931.

## Results and Discussion

### Nanodroplets are a shelf-stable agent that performed better than microbubbles for DNA fragmentation

Microbubbles were initially evaluated as a cavitation enhancement agent in the Covaris E110 sonicator. Microbubbles, however, are rapidly fragmented in a high amplitude acoustic field (half-life in the order of seconds) so they provided effective cavitation enhancement for only 5–10 seconds with the given acoustic parameters. We hypothesized that by condensing microbubbles to form nanodroplets, we could extend the release of microbubbles therefore sustaining the cavitation enhancement.

**Fig 1:**
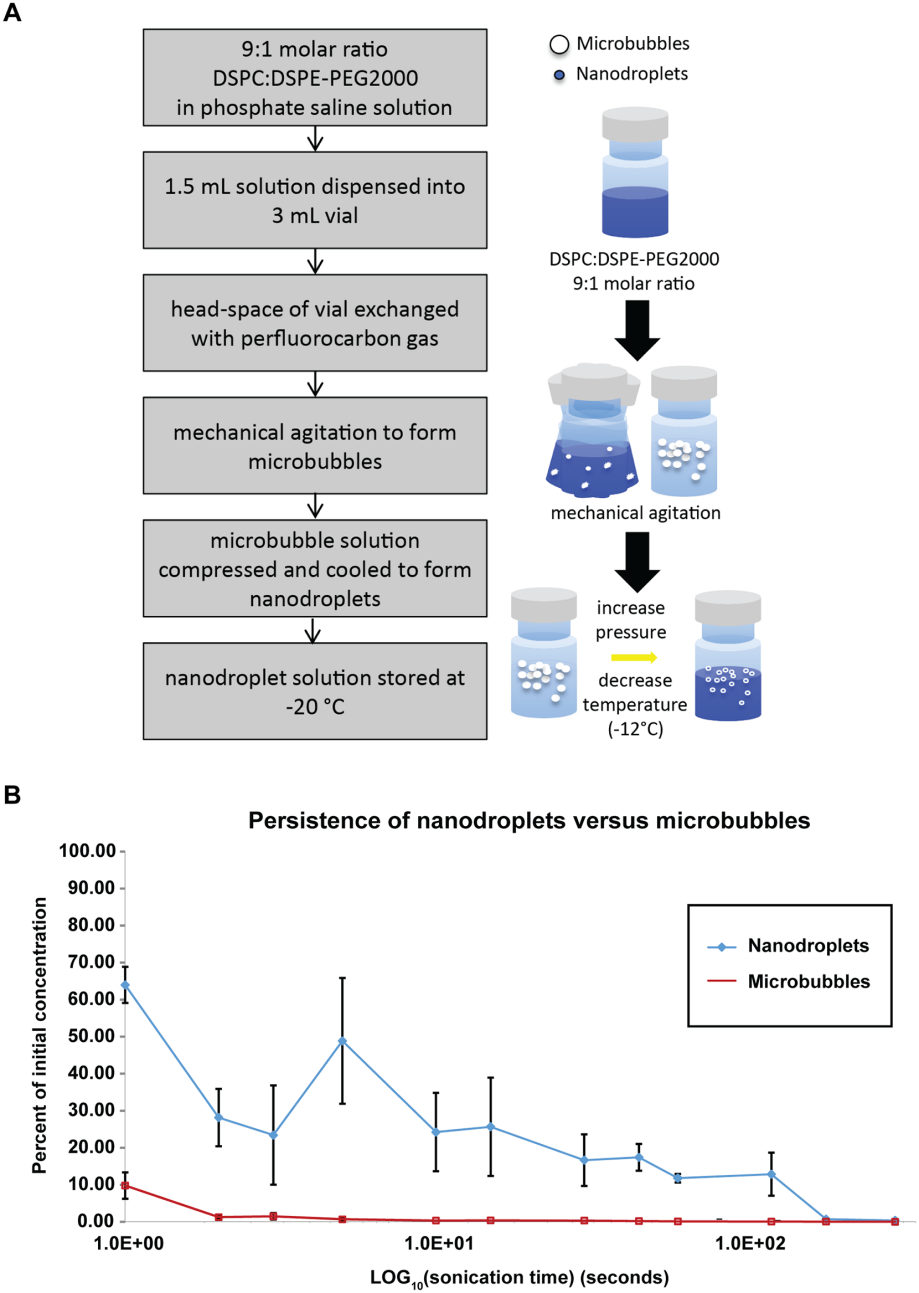
Nanodroplets persisted in solution longer than microbubbles. **A)** Flow chart outlining method for production of nanodroplets. **B)** A persistence study was performed in the ultrasonic bath to compare nanodroplets and microbubbles. An Accusizer particle sizing system (Particle Sizing Systems, Port Richey, FL) was used to measure the microbubble and nanodroplet concentrations at specific time points between 0 and 300 seconds (5 minutes). Nanodroplets maintained between 10–20% of their initial concentration as far out as 3 minutes into the sonication treatment, while the microbubble concentration dropped to 10% after 1 second.

**Fig 2:**
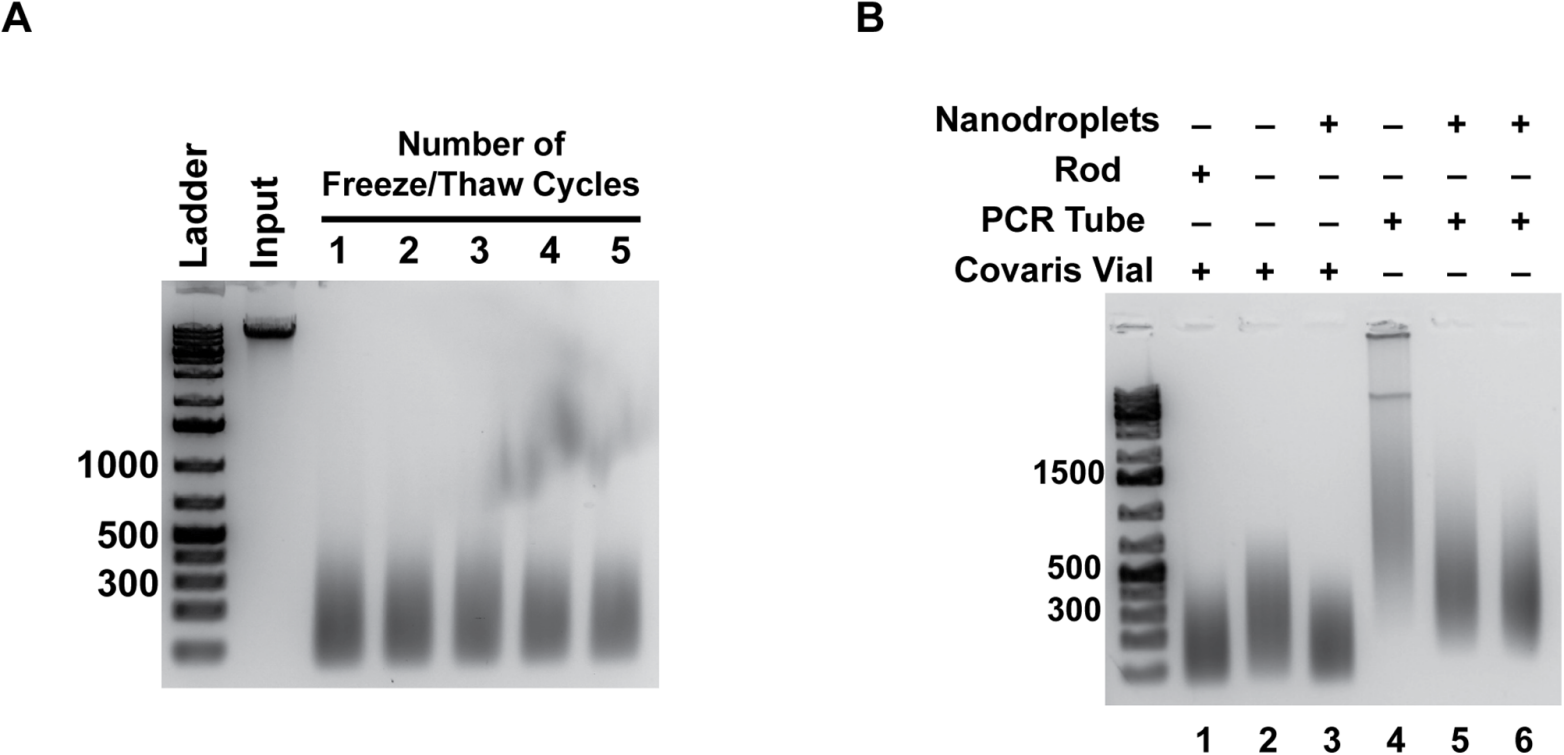
Nanodroplets were an effective cavitation agent for use in DNA fragmentation. **A)** The effectiveness of nanodroplets as a cavitation enhancement agent after multiple freeze-thaw cycles was tested. DNA ladder size is indicated in base pairs. Input is DNA prior to sonication with nanodroplets. **B)** Comparison of DNA fragmentation efficiency after two minutes in glass (Lanes 1–3) versus plastic (Lanes 4–6) tubes in the Covaris E110 sonicator. The addition of nanodroplets to Covaris microTUBES produces a DNA fragment size distribution comparable to the microTUBES used with the supplied rod (compare Lanes 1 and 3). DNA fragmented in glass microTUBES had a smaller DNA size distribution compared to plastic 0.2 mL PCR tubes (compare Lanes 3 and 5–6). DNA ladder size is indicated in base pairs.

Microbubbles were produced through vigorous mechanical agitation of a lipid solution inside a 3 mL vial, followed by simultaneously cooling and increasing ambient pressure of the microbubble solution to condense the bubbles into nanodroplets (Fig 1A). A persistence study was performed to test the half-life of microbubbles versus nanodroplets using the ultrasonic bath, showing that nanodroplets persist much longer with acoustic parameters kept constant (Fig 1B). In addition, we demonstrated that nanodroplets have excellent shelf stability; they can be stored at -20°C with no loss of performance following multiple freeze-thaw cycles (Fig 2A).

### DNA fragments created with nanodroplets in an ultrasonic bath were comparable to those produced with a commercially available method

**Fig 3:**
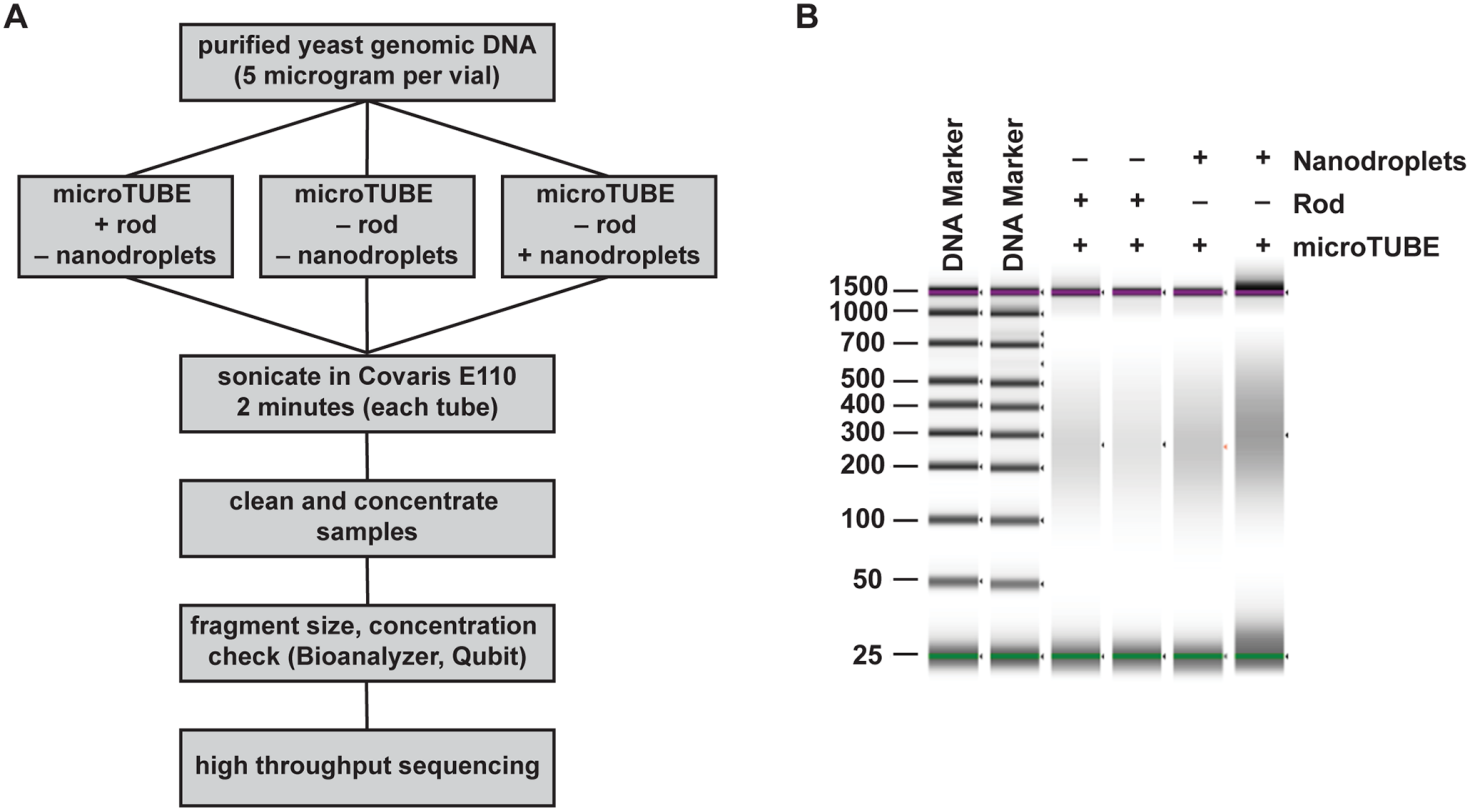
Nanodroplet-mediated DNA fragmentation compared to a commercial method. **(A)** Flow chart outlining method for comparing DNA fragmentation methods. **(B)** False gel picture from Agilent D1000 ScreenTape system showing DNA fragment size distribution in base pairs for samples fragmented in the Covaris E110 sonicator. Purple bars indicate the upper (1,500 bp) molecular weight marker and green bars indicate the lower (25 bp) molecular weight marker in each lane.

DNA fragmentation was initially compared with or without nanodroplets in the commercially available Covaris E110 sonicator. Borosilicate glass vials produced a smaller average fragment size within a given sonication time compared to plastic PCR tubes, so all further experiments in this instrument were performed in the borosilicate glass Covaris microTUBE (Fig 2B, compare lanes 1–3 to lanes 4–6). The Covaris microTUBE contains a hydrophobic polymer rod with small pores that enucleate gas bubbles when exposed to acoustic energy [29]. Comparisons were made between duplicate samples of the microTUBE with the rod, and the microTUBE with nanodroplets (Fig 3A). Genomic DNA purified from the *Saccharomyces cerevisiae* strain, BY4741, was used for this study as the small genome size allowed for high density sequencing coverage.

**Fig 4:**
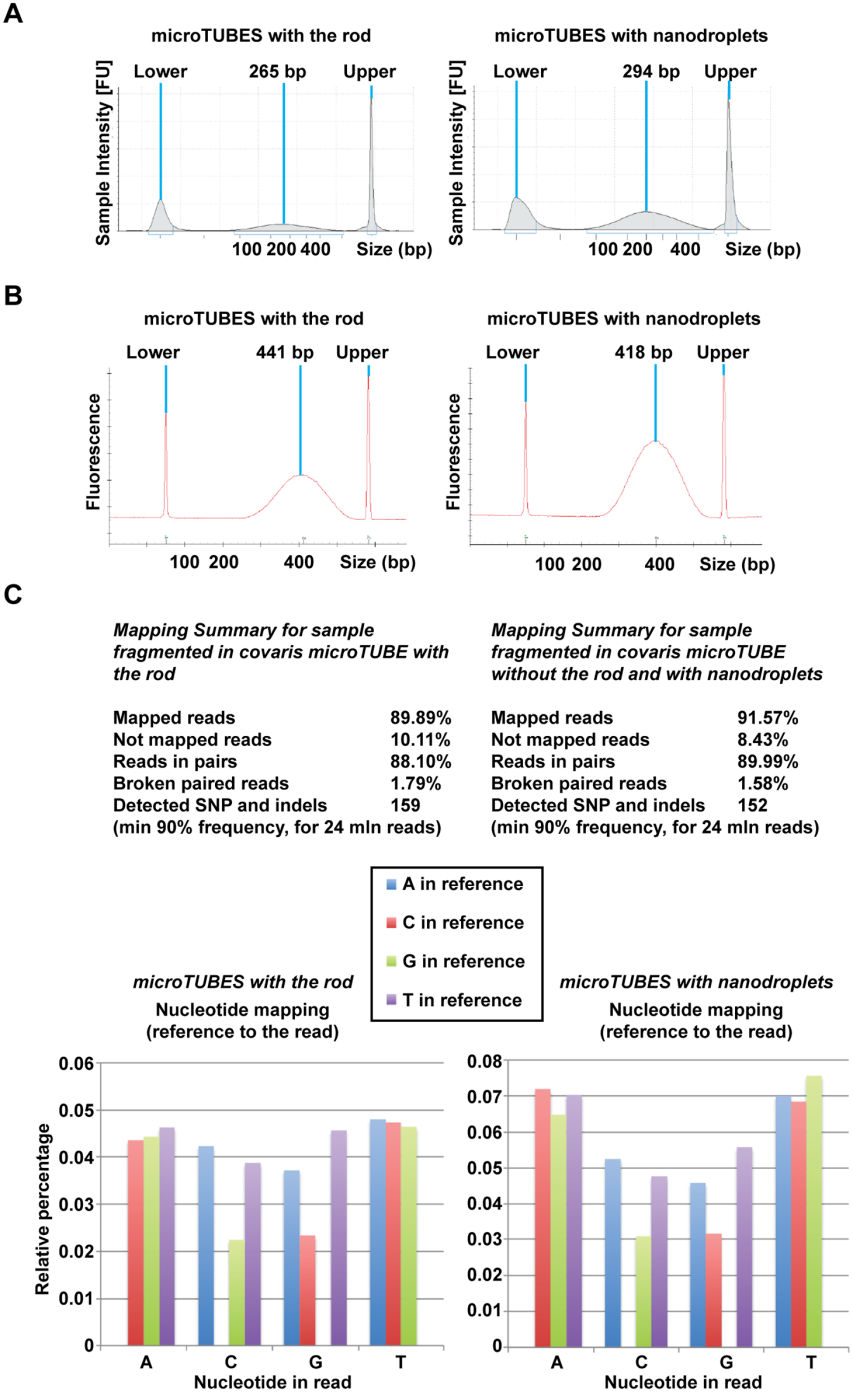
*Saccharomyces cerevisiae* gDNA (BY4741) fragmented with nanodroplets in an ultrasonic bath was comparable in quality to DNA fragmented using a commercial method. **(A)** Agilent D1000 ScreenTape system traces for DNA samples in microTUBES with the rod (left panel) or microTUBES with nanodroplets (right panel) that were subjected to sequencing. Average size is indicated in base pairs (bp). DNA size markers are denoted by Upper and Lower. **(B)** Traces showing similar size distribution of DNA after sequencing library preparation. Average size is indicated in base pairs (bp). DNA size markers are denoted by Upper and Lower. **(C)** Mapping sequencing reads to the *Saccharomyces cerevisiae* (S288c) reference genome is comparable in detection of single nucleotide variations, insertions, and deletions. Abundance and profile of relative errors in sequencing reads does not indicate a difference in the presence of error bias in the data.

Following two minutes of sonication, the average DNA fragment size was comparable between the microTUBE and nanodroplet samples (Figs 3B and 4A). To confirm that the DNA fragmented in the presence of nanodroplets was suitable for downstream applications, we subjected these samples to next-generation sequencing. Following library preparation, no difference in quality and profile was noted between the two samples (Fig 4B) or between duplicate samples. Both average fragment size and the fragment distribution profiles were similar and depended on the chosen size selection method and not sonication method (S1 Table).

Sequencing reads were mapped to the *Saccharomyces cerevisiae* S288C reference genome, from which the BY4741 strain used in this study was derived [30]. Samples fragmented in the presence of nanodroplets showed no appreciable difference in mapped reads, detection of single nucleotide variations and insertions and deletions (indels), or error bias in the data (Fig 4C) compared to samples fragmented in the microTUBEs. Thus, gDNA fragmented in the presence of nanodroplets performed equivalently in high throughput sequencing compared to gDNA fragmented with a commercially available method.

### Nanodroplet-mediated cavitation in a bench top ultrasonic water bath allowed parallel processing of multiple samples

Most commercially available sonication devices are low thoughput, sonicating only a single sample at a time. Since gDNA fragmentation with nanodroplets was effective in a commercially available sonicator, we tested the performance of nanodroplets in a standard laboratory ultrasonic water bath with the aim of increasing sample throughput, and negating the need for specialized equipment.

**Fig 5:**
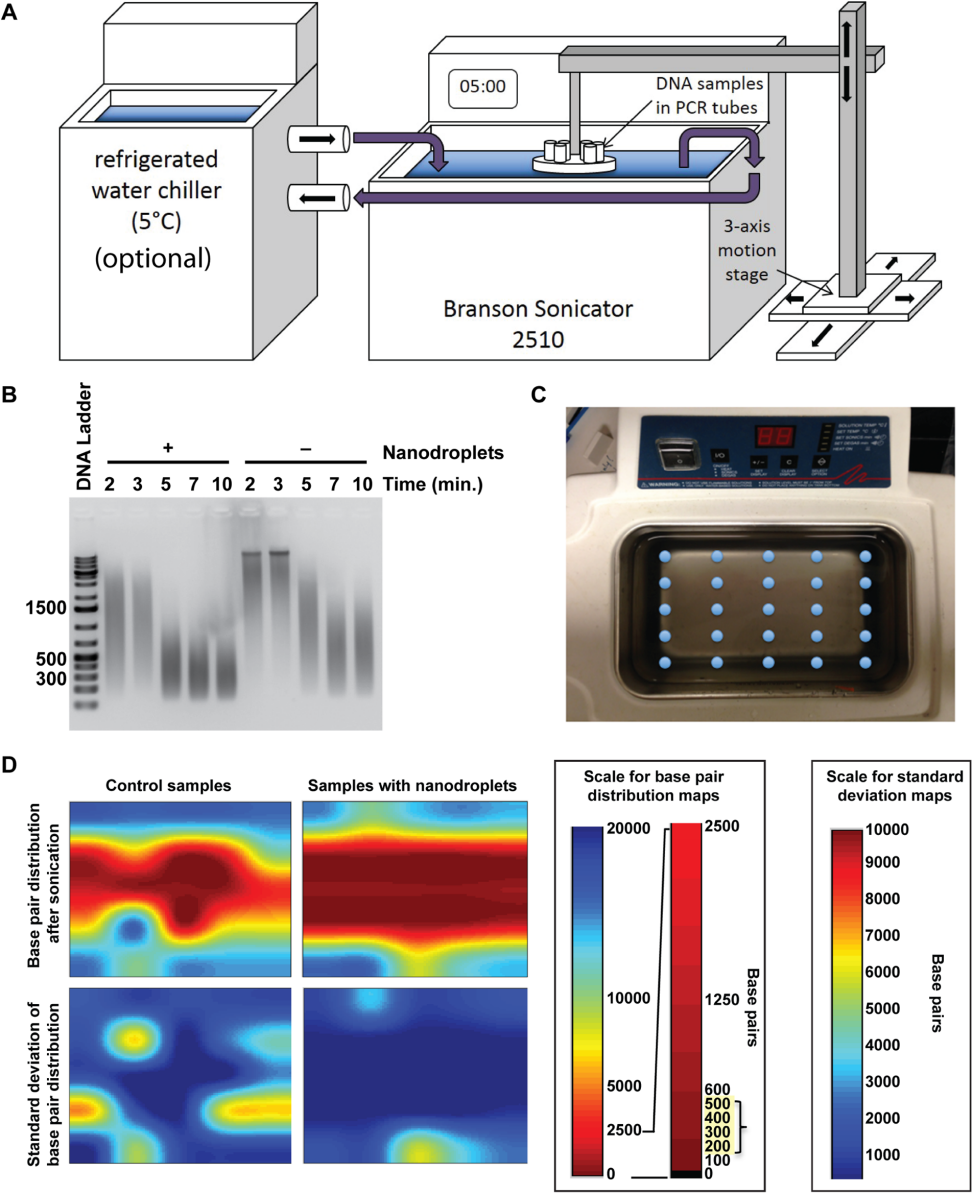
The use of nanodroplets allowed an ultrasonic water bath to fragment genomic DNA. **(A)** Schematic showing the ultrasonic bath used for sonication. Samples were immobilized in the water bath using a stand with a tube rack attached. The circulating water chiller was optional. Water chilled to four degrees Centigrade can be added just prior to sonication, with no loss in DNA fragmentation efficiency. **(B)** A time-titration was performed with samples with and without nanodroplets. Following fragmentation, samples were run on a 1.5% agarose gel and visualized using SYBR green. DNA ladder sizes are indicated in base pairs. **(C)** Arrangement of DNA samples fragmented in the ultrasonic bath with and without samples to produce **(D)** an acoustic field map of the bath. The fragmentation ability (base pair size) is visualized with the s1color bar, where red indicates complete fragmentation in the 200–500 bp range.

A Branson 2510 Ultrasonic Cleaning Bath was converted to a gDNA sonicator by installing a simple custom tube holder to submerge sample vessels at desired positions in the bath. To maintain the temperature conditions of current DNA fragmentation methods, water was pre-chilled to 4°C and added to the ultrasonic bath. Chilled water could also be circulated using a refrigerated water chiller (Fig 5A). The operating level of the ultrasonic bath was demarcated by the manufacturer at double the operating wavelength of the acoustic field in water at room temperature (25°C). To maintain constant acoustic parameters, we adjusted the water level according to the wavelength change of the acoustic field in water at 5°C. Sonication time was optimized to five minutes in thin walled PCR tubes (Fig 5B). These tubes were selected because the thin wall was less likely to interfere with the acoustic field, and they are inexpensive and readily available from a number of commercial vendors. An acoustic field map achieved by sonicating a five-by-five array of DNA samples (Fig 5C) determined that the most consistent gDNA fragmentation occurred in the central region of the bath (Fig 5D), so samples were immobilized within this area using a custom sample holder. The holder for these studies consisted of a single row of 14 samples with 0.5 inch spacing. In future studies, spacing will be optimized further to maximize the number of samples that can be fragmented at once.

**Fig 6:**
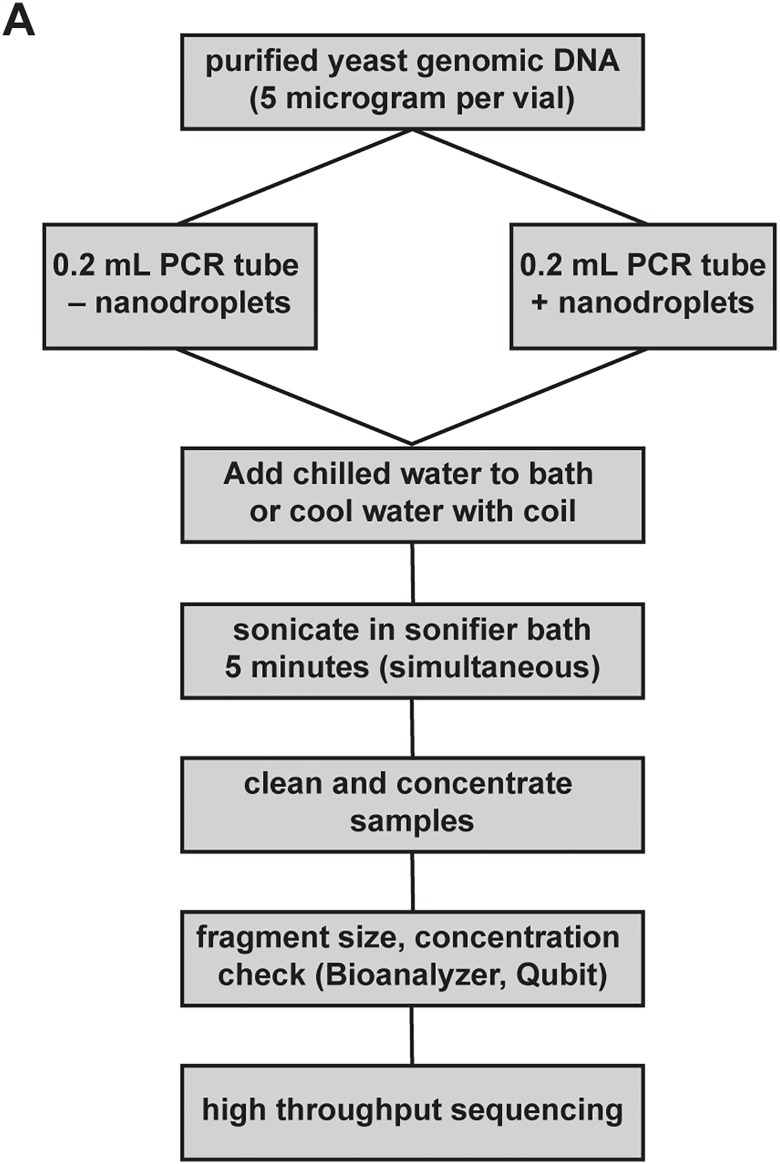
DNA fragmentation in an ultrasonic water bath compared to a commercially available device. **(A)** Flow chart outlining method for comparing DNA fragmentation methods.

**Fig 7:**
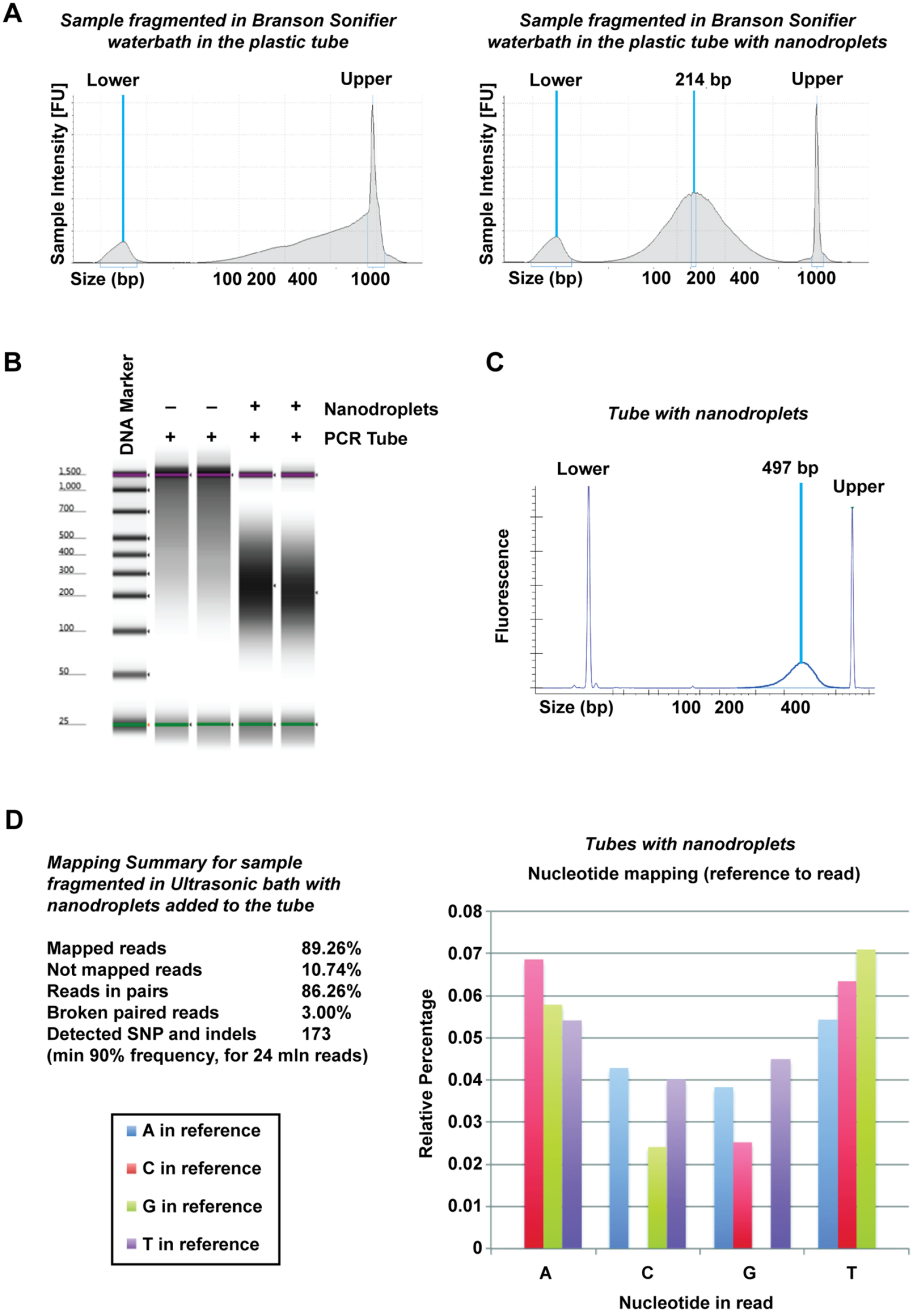
*Saccharomyces cerevisiae* gDNA (BY4741) fragmented with nanodroplets in an ultrasonic water bath was comparable in quality to DNA fragmented in a commercially available device. **(A)** Agilent D1000 ScreenTape data showing size distribution of DNA fragmented in tubes without (left panel) or tubes with nanodroplets (right panel). Average size is indicated in base pairs (bp). DNA size markers are denoted by Upper and Lower. **(B)** False gel picture indicating that DNA fragmented without nanodroplets had an average fragment size >1,500 bp. Purple bars indicate the upper (1,500 bp) molecular weight marker and green bars indicate the lower (25 bp) molecular weight marker in each lane. **(C)** Size distribution of DNA after sequencing library preparation. Average size is shown in base pairs (bp). DNA size markers are denoted by Upper and Lower. **(D)** Mapping sequencing reads to the *Saccharomyces cerevisiae* (S288c) reference genome is comparable in detection of single nucleotide variations and indels in Fig 4C. Abundance and profile of relative errors in sequencing reads does not indicate a difference in the presence of error bias in the data compared to data in Fig 4C.

To confirm that the DNA fragmented in the ultrasonic water bath could be used for next-generation sequencing, fragmentation of BY4741 yeast gDNA was performed in duplicate in the presence and absence of nanodroplets (Fig 6). In the absence of nanodroplets, the average DNA fragment size was >1,500 bp, a size not suitable for library preparation (Fig 7A and 7B, S1 Table). Samples with nanodroplets had an average fragment size of <250 bp before library preparation (Fig 7A and 7B), and an average size of 500 bp after library preparation (Fig 7C and S1 Table), which was comparable to data obtained from fragmentation in the Covaris (Fig 4A and 4B, S1 Table). After sequencing, the duplicate samples fragmented in the presence of nanodroplets showed no appreciable difference in mapped reads, detection of single nucleotide variations and indels, or error bias in the data (Fig 7D). Therefore, cavitation enhancement by nanodroplets in a standard laboratory ultrasonic water bath produced fragmented gDNA that was comparable to DNA obtained from sonication in a commercially available device.

The sonication methods that are employed by the Covaris and the ultrasonic water bath are different with respect to the acoustic frequency used and the distribution of the acoustic field. The Covaris uses a spherically focused 1 MHz ultrasound transducer which produces a very tight (~2 mm) focus. The Branson ultrasonic water bath uses two heavy-duty 40 kHz sandwich type transducers that produce a broad acoustic field within the tank, which is amplified by standing waves created by reflections from the water-air interface and the sides of the tank. DNA can be fragmented using both systems, through the combination of cavitation mechanisms and high microscopic fluid flow (S1 Fig). The overall intensity of the acoustic field in the ultrasonic water tank is less than the acoustic intensity at the focal spot of the Covaris transducer, however the probability threshold for cavitation events increases with decreasing frequency [26]. Furthermore, the addition of the nanodroplets further enhances the cavitation effect. The ultrasonic tank possesses the advantage of increasing the number of cavitation events and having a larger effective acoustic field area allowing greater number of samples to be treated simultaneously (Fig 5D).

## Conclusion

We have demonstrated that fragmentation of gDNA in the presence of our nanodroplet formulation does not require specialized equipment, can be performed for multiple (up to 14) samples simultaneously in five minutes, and produces high quality, fragmented DNA for next-generation sequencing. Phase change nanodroplets are produced using a simple method and can be stored at -20°C for extended periods of time, withstanding multiple freeze-thaw cycles. The use of this cavitation enhancement agent in combination with a standard laboratory ultrasonic bath is a useful and cost-effective method for academic institutions and research laboratories that do not have access to specialized sonication devices, and provides new accessibility and improved efficiency for a crucial step in next-generation sequencing.

## Supporting Information

S1 Fig Starch-Potassium Iodide (Starch-KI) test for cavitation. The starch-iodine test was performed to confirm the presence of cavitation. If cavitation is present, the Starch-KI solution turns from clear to blue. Reactive oxygen species that form during a violent cavitation event transform dissolved iodide ions into iodine. Iodine then conforms to the starch molecule and results in a visible blue color. The results from the Covaris and the ultrasonic bath are displayed in a dot blot array. In both the Covaris and the ultrasonic bath, vials with no rod or nanodroplets showed minimal fragmentation.
(TIF)

S1 Protocol Starch-Potassium Iodide (Starch-KI) test for cavitation. A starch-KI solution (0.01M KI, 3g/L starch) was used to detect the presence of cavitation in both the Covaris and Branson ultrasonic bath setups. In the process of inertial cavitation, the localized release of energy results in the formation of reactive oxygen species (ROS), which react with iodide ions in solution to produce iodine. The iodine intercalates within the starch molecules, staining the starch blue-violet. This simple starch-iodine test is a well-known method of testing for cavitation [31]. 100 μL of starch-KI solution was inserted into borosilicate glass vials for testing in the Covaris. Three conditions were tested; control starch-KI solution without nanodroplets nor rod, starch-KI solution with 10 μL nanodroplets, and starch-KI solution with rod. Each sample was sonicated using the same sonication parameters as described previously (2 minutes, 20% duty cycle, intensity 8, and 200 cycles per burst). For the Branson ultrasonic bath, 150 μL of starch-KI solution was inserted into thin-walled PCR tubes. Samples contained either control starch-KI solution or starch-KI solution with 20 μL nanodroplets. Each sample was sonicated for 5 minutes.
(DOCX)

S1 Table Preparation methods for sequencing libraries. The average fragment size for each sample is shown before and after library preparation. Size selection methods include magnetic bead (Bead) and automatic gel size selection (Pippin Prep, Sage Science). Samples with variable concentrations produced libraries with a similar fragment size.
(DOCX)
