# Supplementary figures and images for: Cavitation Enhancing Nanodroplets Mediate Efficient DNA Fragmentation in a Bench Top Ultrasonic Water Bath
Source: PLoS One. 2015 Jul 17;10(7):e0133014. doi: 10.1371/journal.pone.0133014 (PMC4505845; doi:10.1371/journal.pone.0133014)

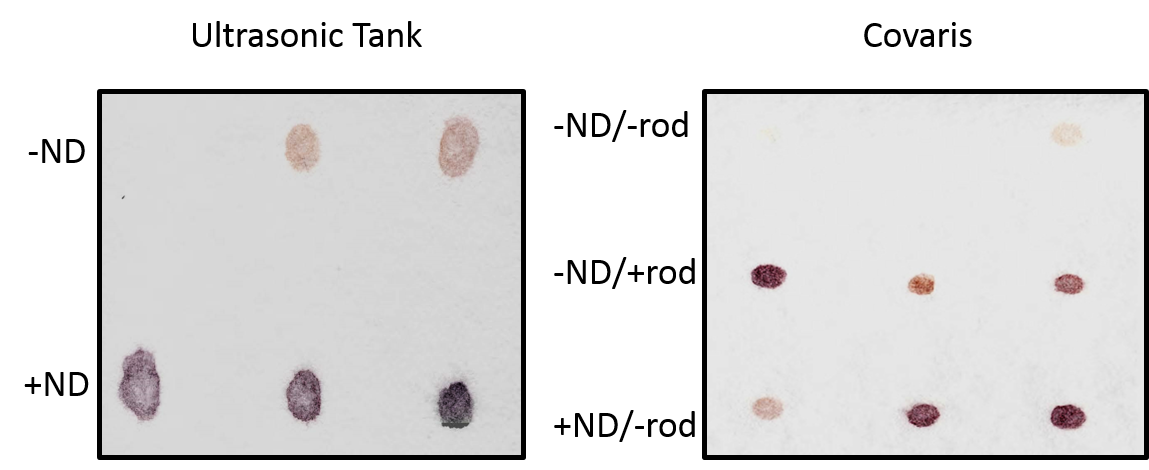

Supplement: S1 Fig — The starch-iodine test was performed to confirm the presence of cavitation. If cavitation is present, the Starch-KI solution turns from clear to blue. Reactive oxygen species that form during a violent cavitation event transform dissolved iodide ions into iodine. Iodine then conforms to the starch molecule and results in a visible blue color. The results from the Covaris and the ultrasonic bath are displayed in a dot blot array. In both the Covaris and the ultrasonic bath, vials with no rod or nanodroplets showed minimal fragmentation. (TIF) [file pone.0133014.s001.tif]
