# Supplementary material for: Cavitation Enhancing Nanodroplets Mediate Efficient DNA Fragmentation in a Bench Top Ultrasonic Water Bath
Source: PLoS One. 2015 Jul 17;10(7):e0133014. doi: 10.1371/journal.pone.0133014 (PMC4505845; doi:10.1371/journal.pone.0133014)
Supplement: S1 Protocol — A starch-KI solution (0.01M KI, 3g/L starch) was used to detect the presence of cavitation in both the Covaris and Branson ultrasonic bath setups. In the process of inertial cavitation, the localized release of energy results in the formation of reactive oxygen species (ROS), which react with iodide ions in solution to produce iodine. The iodine intercalates within the starch molecules, staining the starch blue-violet. This simple starch-iodine test is a well-known method of testing for cavitation [31]. 100 μL of starch-KI solution was inserted into borosilicate glass vials for testing in the Covaris. Three conditions were tested; control starch-KI solution without nanodroplets nor rod, starch-KI solution with 10 μL nanodroplets, and starch-KI solution with rod. Each sample was sonicated using the same sonication parameters as described previously (2 minutes, 20% duty cycle, intensity 8, and 200 cycles per burst). For the Branson ultrasonic bath, 150 μL of starch-KI solution was inserted into thin-walled PCR tubes. Samples contained either control starch-KI solution or starch-KI solution with 20 μL nanodroplets. Each sample was sonicated for 5 minutes. (DOCX) [file pone.0133014.s002.docx]

**Supporting Information**

**Protocol**

**S1 Protocol. Starch-Potassium Iodide (Starch-KI) test for cavitation.** A starch-KI solution (0.01M KI, 3g/L starch) was used to detect the presence of cavitation in both the Covaris and Branson ultrasonic bath setups. In the process of inertial cavitation, the localized release of energy results in the formation of reactive oxygen species (ROS) which react with iodide ions in solution to produce iodine. The iodine intercalates within the starch molecules, staining the starch blue-violet. This simple starch-iodine test is a well-known method of testing for cavitation [35].

100 μL of starch-KI solution was inserted into borosilicate glass vials for testing in the Covaris. Three conditions were tested; control starch-KI solution without nanodroplets nor rod, starch-KI solution with 10 μL nanodroplets, and starch-KI solution with rod. Each sample was sonicated using the same sonication parameters as described previously (2 minutes, 20% duty cycle, intensity 8, and 200 cycles per burst).

For the Branson ultrasonic bath, 150 μL of starch-KI solution was inserted into thin-walled PCR tubes. Samples contained either control starch-KI solution or starch-KI solution with 20 μL nanodroplets. Each sample was sonicated for 5 minutes.

**Supplemental Figures and Tables**

**S1 Figure.** **Starch-Potassium Iodide (Starch-KI) test for cavitation.** The starch-iodine test was performed to confirm the presence of cavitation. If cavitation is present, the Starch-KI solution turns from clear to blue. Reactive oxygen species that form during a violent cavitation event transform dissolved iodide ions into iodine. Iodine then conforms to the starch molecule and results in a visible blue color. The results from the Covaris and the ultrasonic bath are displayed in a dot blot array. In both the Covaris and the ultrasonic bath, vials with no rod or nanodroplets showed minimal fragmentation.

**S1 Table. Preparation methods for sequencing libraries.** The average fragment size for each sample is shown before and after library preparation. Size selection methods include magnetic bead (Bead) and automatic gel size selection (Pippin Prep, Sage Science). Samples with variable concentrations produced libraries with a similar fragment size.
