# Supplementary material for: Cavitation Enhancing Nanodroplets Mediate Efficient DNA Fragmentation in a Bench Top Ultrasonic Water Bath
Source: PLoS One. 2015 Jul 17;10(7):e0133014. doi: 10.1371/journal.pone.0133014 (PMC4505845; doi:10.1371/journal.pone.0133014)
Supplement: S1 Table — The average fragment size for each sample is shown before and after library preparation. Size selection methods include magnetic bead (Bead) and automatic gel size selection (Pippin Prep, Sage Science). Samples with variable concentrations produced libraries with a similar fragment size. (DOCX) [file pone.0133014.s003.docx]

| **Sonication Device** | **Sample ID** | **Average Fragment Size (bp)** | **Concentration (ng/μL)** | **Size Selection Method** | **Average Fragment Size After Library Preparation (bp)** |
| --- | --- | --- | --- | --- | --- |
| Covaris | microTUBE with rod | 265 | 7.26 | Pippin Prep | 480 |
| Covaris | microTUBE with rod | 267 | 20.4 | Bead | 440 |
| Covaris | microTUBE with nanodroplets | 259 | 12 | Pippin Prep | 470 |
| Covaris | microTUBE with nanodroplets | 294 | 35.8 | Bead | 420 |
| Ultrasonic Bath | PCR tube no additive | >1,500 | N/A | N/A | N/A |
| Ultrasonic Bath | PCR tube no additive | >1,500 | N/A | N/A | N/A |
| Ultrasonic Bath | PCR tube with nanodroplets | 246 | 24.6 | Bead | 503 |
|  |  |  | 15.6 | Bead | 515 |
| Ultrasonic Bath | PCR tube with nanodroplets | 214 | 32.2 | Bead | 497 |
|  |  |  | 9.4 | Bead | 482 |
